# Corticofugal Augmentation of the Auditory Brainstem Response With Respect to Cortical Preference

**DOI:** 10.3389/fnsys.2019.00039

**Published:** 2019-08-21

**Authors:** Xiuping Liu, Oliver Zhang, Amber Chen, Kaili Hu, Günter Ehret, Jun Yan

**Affiliations:** ^1^Department of Physiology and Pharmacology, Hotchkiss Brain Institute, Cumming School of Medicine, University of Calgary, Calgary, AB, Canada; ^2^Institute of Neurobiology, University of Ulm, Ulm, Germany

**Keywords:** ABR, cochlear enhancement, corticofugal modulation, descending auditory system, focal conditioning, frequency-specific enhancement, mouse, primary auditory cortex

## Abstract

Physiological studies documented highly specific corticofugal modulations making subcortical centers focus processing on sounds that the auditory cortex (AC) has experienced to be important. Here, we show the effects of focal conditioning (FC) of the primary auditory cortex (FC_AI_) on auditory brainstem response (ABR) amplitudes and latencies in house mice. FC_AI_ significantly increased ABR peak amplitudes (peaks I–V), decreased thresholds, and shortened peak latencies in responses to the frequency tuned by conditioned cortical neurons. The amounts of peak amplitude increases and latency decreases were specific for each processing level up to the auditory midbrain. The data provide new insights into possible corticofugal modulation of inner hair cell synapses and new corticofugal effects as neuronal enhancement of processing in the superior olivary complex (SOC) and lateral lemniscus (LL). Thus, our comprehensive ABR approach confirms the role of the AC as instructor of lower auditory levels and extends this role specifically to the cochlea, SOC, and LL. The whole pathway from the cochlea to the inferior colliculus appears, in a common mode, instructed in a very similar way.

## Introduction

Species-specific (evolutionary) adaptations and individual-specific adjustments (experience and learning) are integrated in the responsiveness of the auditory cortex (AC) to a given sound (Ehret, 1997; Scheich and Ohl, 2011; Weinberger, 2011; Geissler et al., 2016). If the sound has proven to be important, the AC has been found to feedback to the ascending auditory system including the cochlea (CO) to improve the processing of this sound. The feedback is conveyed by multiple corticofugal pathways, including direct projections from the AC to the auditory midbrain (inferior colliculus, IC), lateral lemniscus (LL), superior olivary complex (SOC), and cochlear nucleus (CN) and indirect projections to the CN and CO *via* the IC and the SOC (Spangler and Warr, 1991; Feliciano et al., 1995; Winer, 2006; Malmierca and Ryugo, 2011; Schofield, 2011; Schofield and Beebe, 2018). Many studies have suggested that this feedback of a conditioned/experienced AC may lead to changes in tonotopy, sharpness of frequency tuning, response threshold, response strength, response latency, dynamic range, and directional sensitivity at subcortical processing centers (Zhang et al., 1997; Xiao and Suga, 2002; Yan and Ehret, 2002; Suga and Ma, 2003; Yan et al., 2005; Perrot et al., 2006; Zhou and Jen, 2007; Luo et al., 2008; Xiong et al., 2009; Liu et al., 2010, 2019; Suga, 2012; Bajo and King, 2013; Kong et al., 2014; Aedo et al., 2016). These subcortical changes reflect substantial corticofugal impact. Many aspects of this impact, although essential for understanding the function and role of the corticofugal pathways as the whole and in detail, are still unknown. For example, the possible physiological impact on the LL has not yet been studied at all. Also unknown are the absolute and relative amounts of the corticofugal influence on the auditory responses at each processing level from the cochlea upwards. In other words, it remains to be shown whether corticofugal effects just add up from the cochlea onwards to arrive as a predictable sum of effects at the cortical level or are newly and specifically created at each processing level.

In this study, we intend to address the above issues by examining both absolute and relative changes in the peak amplitudes and latencies of auditory brainstem responses (ABR) due to AC conditioning. The ABR technique allows simultaneous recording and evaluation of synchronized neural responses from various centers along the ascending auditory pathways (Eggermont and Schmidt, 1990; Hall, 2007). The ABR often has five wave peaks. In the mouse, peak one (PI) is related to responses of the cochlea and auditory nerve, peaks two, three, four, and five (PII–PV) represent mainly responses of cell groups in the CN ipsilateral to the stimulated ear, in the contralateral SOC, in the contralateral LL and IC, respectively (Henry, 1979a,b; Parham et al., 2001; Land et al., 2016). Thus, ABR amplitudes and latencies recorded before and after AC focal conditioning (FC) to a given sound provide the measures for simultaneously examining the amount of corticofugal influence on different processing levels in a single experimental approach.

## Materials and Methods

### General

Animal preparation and FC of the mouse primary auditory cortex (AI) with pairs of tones and electric pulses (focal conditioning of the primary auditory cortex, FC_AI_) have been described before (Yan and Ehret, 2002; Yan et al., 2005). Nine female house mice (*Mus domesticus*, outbred strain, NMRI) aged 2–3 months and weighing 25–34 g were used. Animals were anesthetized with a mixture of ketamine (Ketavet, 120 mg/kg, ip) and xylazine (Rompun, 5 mg/kg, ip). Additional dosages of 25 mg/kg ketamine + 1 mg/kg xylazine were administered about every 40 min in order to maintain the anesthetic level during the experiment. The animal’s head was immobilized in a custom-made head holder by clamping the palate and nasal/frontal bones. The mouth bar was adjusted to align Bregma and lambda points of the skull in one horizontal plane. The scalp overlaying the dorsal skull was removed and two holes with a diameter of 2 mm were drilled, one in the temporal bone covering the left-side AC and the other in the middle of the occipital bone 1 mm posterior to the lambda point. During and after surgery, the animal was placed on a feedback-controlled heating pad at 37°C. At the end of an experiment, the still anesthetized animal was killed by cervical dislocation. The animal protocol was in accordance with the European Communities Council Directive (86/609/EEC) and approved by the appropriate authority (Regierungspräsidium Tübingen, Germany).

### Acoustic Stimulation

The measurements took place in an electrically shielded, sound-attenuated, and anechoic (in the frequency range of interest) chamber. Tone bursts (20 ms long with 1 ms rise-decay time) were generated by a voltage-controlled frequency generator (Wavetek 193) and an electronic switch. Tone frequency was altered by various levels of DC voltages, generated with a CED 1401plus (CED Inc., England) and fed to the VCG IN of the frequency generator. Tone amplitude was altered by an attenuator (Kenwood RA 920A). The tone bursts were sent *via* a power amplifier (Denon PMA 1060) to a dynamic speaker (Thiel C2 33/8) and *via* a voltage amplifier (Hewlett-Packard, 465A) and power supply to an electrostatic speaker (Machmerth et al., 1975). Both loudspeakers were placed 45° lateral to and 40 cm away from the animal’s right ear. The interval between tone bursts was 500 ms. Sound pressure, expressed in decibel sound pressure level (dB SPL), was calibrated at the right ear of the animal with a Bruel and Kjaer condenser microphone (type 4135) and a measuring amplifier (type 2636). Acoustic stimulation was controlled by an electric pulse generated with the CED 1401plus and Spike2 data acquisition system (CED Inc., England) and visualized on an oscilloscope (Tektronix 2216).

### Neural Recording From and Conditioning of the AI

In order to define the tonotopic place of the intended FC_AI_, a tungsten electrode (WPI, TM33C20KT) with a tip impedance of ~2 MΩ was placed on the suggested tonotopic map of the left AI (Stiebler et al., 1997) perpendicularly to the AI surface and advanced to a depth of ~400 μm where tone-evoked multi-unit responses were most robust. For the recordings, the electrode output was amplified (10,000 times, WPI DAM 80 preamplifier), band-pass filtered (0.3–10 kHz) and fed to the oscilloscope and to an audio monitor. Once the recording was stable, the best frequency (BF) and minimum threshold (MT) of the recorded AI neurons were measured by varying frequencies and SPLs of the tone bursts. When a suitable BF and MT were found, the electrode was advanced to a depth of ~700 μm below the brain surface (layer V) and the connection was switched to the input of a constant current isolator (WPI 360) to provide electrical stimulation. An indifferent electrode was placed on the brain surface just beside the stimulating electrode. One-ms-long monophasic electrical pulses with 500-nA constant negative current were created by the WPI A360 and the CED 1401plus. The current of the stimulating electrode was synchronized with the offset of BF tone bursts presented at 20 dB above MT of the stimulated neurons. Thus, the cortical response to every tone stimulus was reinforced by a presumably strong response to the electric pulse. The pair of tone burst and electric pulse was delivered at a rate of 4 Hz for 7 min. This contingency of the tonal and electrical stimulation is comparable to a conditioning paradigm using the electrical pulse as an unconditioned stimulus evoking a cortical response and the tone burst as the stimulus to be conditioned (conditioned stimulus). We used this stimulation paradigm of AI because it reproduced those in our previous studies (Yan and Ehret, 2001, 2002; Yan et al., 2005) in which the tonotopy and neuronal rate-level functions, response thresholds and frequency tunings in the IC were specifically changed after FC_AI_.

### Recording of the ABR

The first set of ABR data was taken prior to the FC_AI_ with the pairs of tone bursts and electrical pulses, the second set of data was taken with identical acoustical stimulation during a 1–3 h period after the end of the FC_AI_ when FC_AI_ effects have been shown to be the largest in the IC (Yan and Ehret, 2001). Two silver wires (diameter 0.25 mm) were used as ABR recording electrodes. One electrode (active) was placed on the dura mater of the vertex, 1 mm posterior to the lambda point. The other electrode (reference) was subcutaneously placed just below the pinna of the right ear. ABR potentials evoked by tones of 20 ms duration (1 ms rise-decay times included) were amplified 10,000 times, filtered with a bandpass of 0.2–5 kHz and then fed into the CED1401plus for data acquisition and analysis (Spike2 software). In the ABR recordings, five-tone frequencies were used, tones at the BF that has been determined before, and at 5 kHz and 15 kHz higher and lower than the BF, respectively. The SPLs of the tones were set at 65, 45, 25, 15, 10, and 5 dB SPL, respectively. For each stimulus of a given frequency and SPL, data were collected over 20 ms from the tone onset. Responses to 500 tone bursts with 500 ms inter-tone were averaged to obtain the ABR data.

### Data Processing

**Figure 1:**
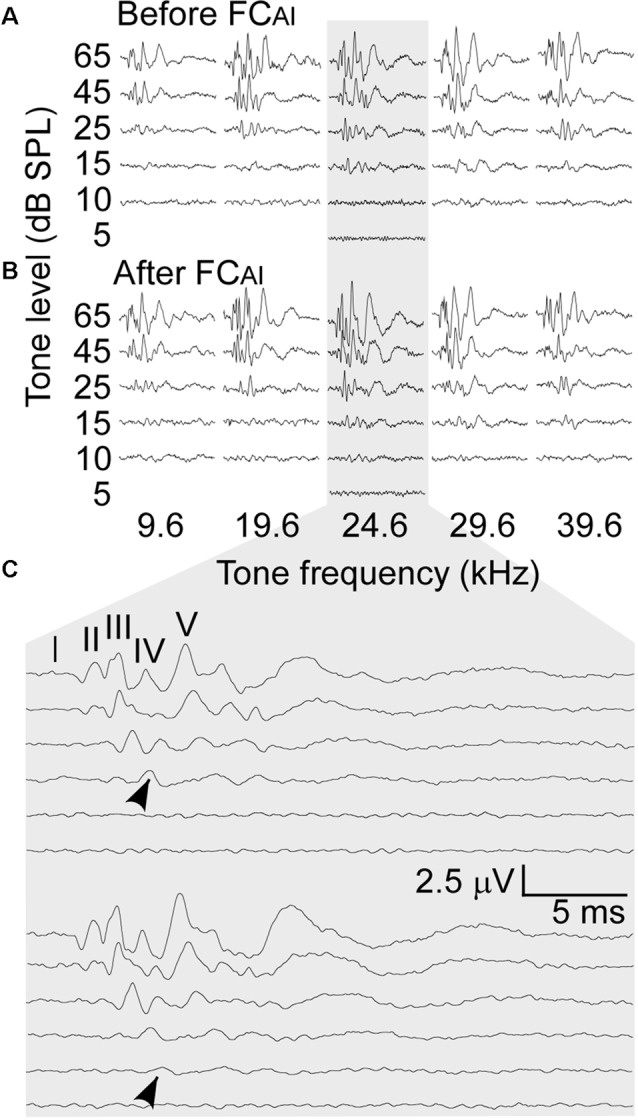
An example of the auditory brainstem responses (ABRs) to various tone frequencies and amplitudes before and after focal conditioning (FC) of the primary auditory cortex (AI; FC_AI_). **(A)** The ABRs before FC_AI_. **(B)** The ABRs after FC_AI_. **(C)** Expanded ABRs to the tone of 24.6 kHz that was the best frequency (BF) of the stimulated AI neurons. Arrows indicate the amplitudes taken as ABR thresholds.

The ABR waves were displayed on the computer screen and identified visually. Waves III and V were most prominent and present even when the stimulus tones were of low intensity (Figure 1). Therefore, we distinguished these two waves first and then determined others according to the interpeak latencies known from other ABR studies in mice (Henry, 1979a,b; Kurt et al., 2009; Geissler et al., 2018). The latency and amplitude of each wave were measured with x- and y-cursors of Spike2 software. The wave latency was defined as the time from the onset of the tone burst to the positive peak of each wave. The amplitude of each wave was derived as the difference between the positive peak to the following negative one in the valley of the wave. Many effects of FC_AI_ were established by comparing ABR parameters before and after FC_AI_ (paired *t*-test). Statistical significance was considered in two-tailed tests with the significance level of *α* set at 0.05.

## Results

**Figure 2:**
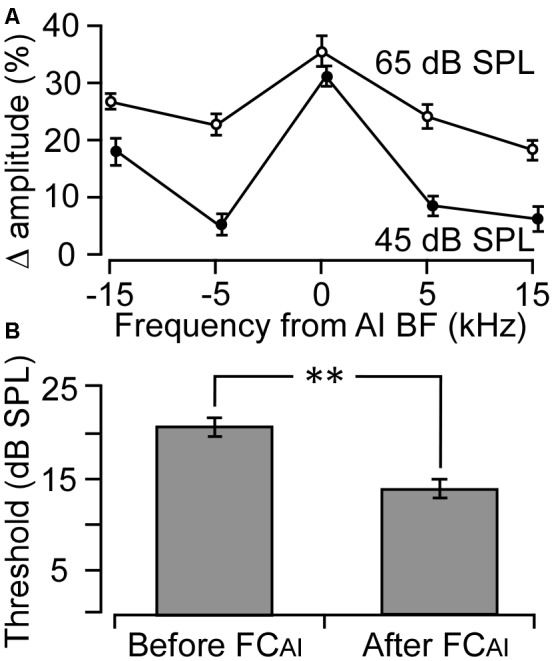
Changes in ABR amplitude and threshold following FC of the AI (FC_AI_). **(A)** The average percentage changes in ABR amplitude to five different frequencies deviating from the AI BF. Zero indicates that the tone frequency was equal to the BF of the stimulated AI neurons. **(B)** Average ABR thresholds before and after FC of the AI. **p < 0.01. Error bars are standard error.

The effects of FC_AI_ on ABRs were examined in nine mice. The BFs of the stimulated AI neurons ranged from 19.6 kHz to 31.5 kHz, with an average of 24.3 ± 3.28 kHz, which falls into the central low-threshold BF range of the NMRI mouse AI (Joachimsthaler et al., 2014). As shown in Figure 1, ABRs showed clear peaks in response to all five tone frequencies used for stimulation when the tone level was at 45 or 65 dB SPL. The amplitudes of the peaks generally increased after FC_AI_. We quantified this amplitude increase as percentage increase averaged together from PIII and PV amplitudes, separately at the tone levels of 45 and 65 dB (Figure 2A). The largest relative increases were noted when the stimulating tone was at the BF of the activated AI locus and the SPL was at 45 dB. FC_AI_ also decreased ABR thresholds, in the shown example (Figure 1) from 15 dB to 10 dB SPL for 24.6 kHz tones (BF of the activated AI locus). ABR thresholds were estimated from the lowest SPL at which PIII and/or PV could be distinguished from the recorded noise background. On average, ABR thresholds for the tone frequency set at the BF of the activated AI locus were 20.6 ± 1.86 dB before and 14.4 ± 1.64 dB SPL after FC_AI_, which is a significant (*p* < 0.01) threshold decrease of 6.2 dB (Figure 2B).

This general analysis indicated that tones of 45 dB SPL were sufficient to induce the ABR peaks and that the largest relative increase in ABR amplitude following FC_AI_ was at the frequency equal to the BFs of stimulated AI neurons. Therefore, we selected the ABRs evoked by tones with frequencies at AI BF and a level of 45 dB SPL for further analyzing the effects of FC_AI_ on latencies and amplitudes of the five ABR peaks.

**Figure 3:**
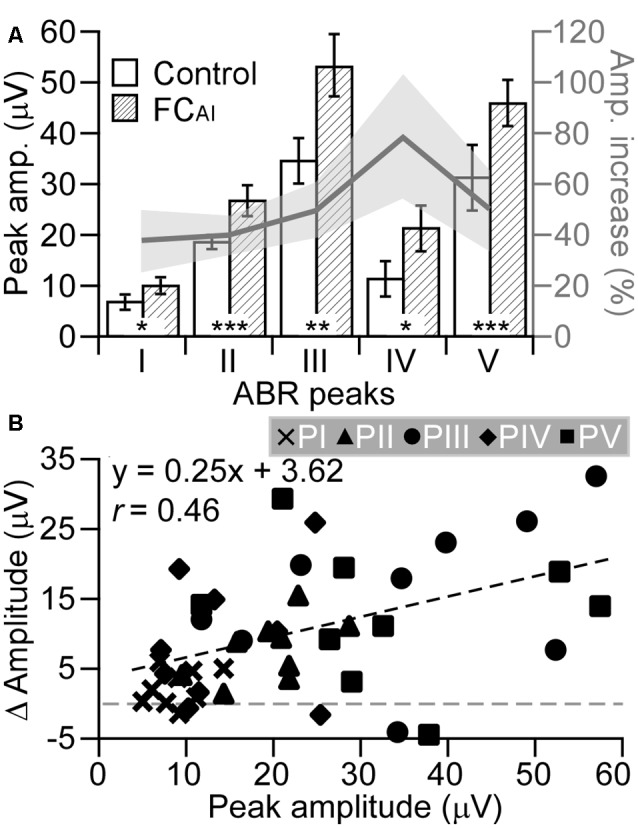
Changes of peak amplitudes of the five ABR peaks (I, II, III, IV, V) following FC of the AI (FC_AI_). **(A)** The absolute amplitudes of all five peaks before and after FC_AI_ (left ordinate) and the percent amplitude increase after FC_AI_ (gray line with standard error, right ordinate). **(B)** The relationship of the changes in peak amplitudes (*y*-axis) due to FC_AI_ to the control amplitudes before FC_AI_. Values of all nine experimental animals at all five peaks are shown. The regression line indicates a significant relationship (*p* < 0.002). *, ** and *** indicate the statistical significance of *p* < 0.05, *p* < 0.01 and *p* < 0.001, respectively. Errors bars and gray area in **(A)** represent standard error.

The ABR amplitudes at the five peaks significantly increased by FC_AI_ as shown in Figure 3A. The increases in the absolute values of the peak amplitudes due to FC_AI_ were tested for significant differences between the peaks. An analysis of variance (ANOVA) on ranks (*DF* = 4; *H* = 11.733; *p* = 0.019) indicated such differences. Pairwise comparisons (*t*-test or *U*-test with Bonferroni correction for repeated testing considered) of the amplitude increases at the peaks showed that the amplitude increase at PI was significantly smaller than that at PII (*p* < 0.05), PIII (*p* < 0.05), and PV (*p* = 0.05). There were no other significant differences between absolute amplitude increases of the peaks. Figure 3A also shows average relative amplitude increases from the values of the unconditioned case. The increases were 35% at PI, 40% at PII, 50% at PIII, 78% at PIV, and 49% at PV. The rather constant relative amplitude increase of average 44% at peaks I, II, III, and V suggested that the absolute amplitude change due to FC_AI_ was a function of the peak amplitude before FC_AI_. Despite the large scatter of the data points from all animals and all peaks (Figure 3B), the plotted regression line (*y* = 0.255x + 3.627) indicated a significant relationship (*N* = 45, *r* = 0.463, *p* < 0.002). In Figure 3B, the dense clustering (small variation) of the data points from PI at small values is clearly visible showing again the difference in data variation between PI and the other peaks.

The latencies of the ABR peaks increased from average 1.89 ms at PI to 6.42 ms at PV, resulting in a 4.53 ms latency increase of activation from the cochlea to the IC (Table 1). After FC_AI_, the total average latency increase from PI to PV amounted to 4.45 ms, a reduction of 0.08 ms compared to the control condition before FC_AI_ (Table 1). We found very similar average latency decreases due to FC_AI_ already at PI (0.09 ms), PII (0.05 ms), PIII (0.10 ms), and PIV (0.08 ms; Table 1). At PV, the latency decrease due to FC_AI_ was about twice as large (0.17 ms) as at the cochlear and lower brainstem levels (Table 1). Therefore, the latency decrease of 0.08 ms due to FC_AI_ in the whole pathway from the CO to the midbrain is reproduced by very similar latency decreases at the cochlea and each following level of the auditory pathway except the IC.

**Table 1:** Latencies ± SD (ms) at the five wave peaks (PI, PII, PII, PIV, PV) and the latency difference PV − PI before and after FC_AI_.

	Before	After	Difference	*p*-value
PI	1.89 ± 0.17	1.80 ± 0.17	0.09 ms = 4.76%	0.044
PII	2.70 ± 0.09	2.65 ± 0.09	0.05 ms = 1.85%	0.003
PIII	3.66 ± 0.16	3.56 ± 0.11	0.10 ms = 2.73%	0.037
PIV	4.86 ± 0.35	4.78 ± 0.37	0.08 ms = 1.65%	0.143^ns^
PV	6.42 ± 0.53	6.25 ± 0.58	0.17 ms = 2.65%	0.015
PV − PI	4.53	4.45	0.08 ms	

*Also, absolute and relative latency differences (before − after) and the significance of the latency changes (*p*-value) are indicated. FC_AI_, focal conditioning of the primary auditory cortex, AI; SD, standard deviation; ns, non-significant*.

In summary, the analysis of ABR peak amplitudes has shown that FC_AI_ increased the amplitudes most when the tone stimulus matched the BF of the conditioned locus at the AI tonotopy. The amplitudes of all five ABR peaks increased significantly with the absolute and relative smallest increase at PI and correlated with the peak amplitudes measured before FC_AI_, except at PIV. FC_AI_ shortened the average latency of each ABR peak by small but significant (for peaks I, II, III, V) and peak-specific amounts.

## Discussion

Corticofugal modulation of sound information processing in the lower auditory centers CO, CN, and IC of bats and mice has been shown to be highly frequency-specific (Zhang et al., 1997; Zhang and Suga, 2000; Yan and Ehret, 2001, 2002; Xiao and Suga, 2002; Yan et al., 2005; Zhou and Jen, 2007; Luo et al., 2008; Liu et al., 2010, 2019; Kong et al., 2014). This frequency specificity was present also in our ABR data (Figures 1, 2A). Since our data analysis was restricted to the ABR to AC-conditioned frequencies, the discussion of our data will be focused on the effects of FC_AI_ with this frequency match. Corticofugal influences in this study were characterized by the increases of ABR amplitudes and decreases of ABR thresholds (Figures 1–3), and shortening of the latencies of the ABR wave peaks (Table 1). These changes in peaks I–V (Figure 3, Table 1) and their implications will be discussed below.

At the cochlear (CO) level, FC_AI_ significantly shortened the PI latency by an average of 0.09 ms (Table 1). This is 9% of the 1 ms rise time of our tone stimulus. This means that the tone-response threshold of the auditory nerve fibers could be reached 0.09 ms earlier after FC_AI_ compared to the control condition (before FC_AI_). On the linear scale from 0 dB to 45 dB over 1 ms rise time, the 9% advance could be equivalent to 4.05 dB decrease of the response threshold if the SPL change and threshold change were linearly related during this period. Thus, the 0.09 ms PI latency decrease suggests about 4 dB increase in CO sensitivity to the frequency that was used for AC conditioning. This positive effect of FC_AI_ on the contralateral cochlear sensitivity may not be mediated by the medial olivocochlear (MOC) system because its activation (either directly or indirectly) reduces contralateral cochlear compound action potential (CAP) amplitudes (Desmedt and Monaco, 1961; Mulders and Robertson, 2000; Groff and Liberman, 2003; Guinan, 2005; Elgueda et al., 2011) and consequently leads to a reduction of the PI amplitude of the ABR (Burkard et al., 1993; Parham et al., 2001). The MOC system acts on cochlear outer hair cells so that modulation (mostly reduction) of cochlear microphonics and otoacoustic emissions that directly relate to the function of outer hair cells have been interpreted as immediate effect of activation of the MOC system *via* auditory cortical or subcortical stimulation (for reviews, see Terreros and Delano, 2015; Lopez-Poveda, 2018; Schofield and Beebe, 2018). Therefore, a *decrease* of CAP and ABR PI amplitudes due to MOC activation would be the result of sensitivity decrease at the level of inner hair cells mediated by outer hair cell function. Hence, the significant PI amplitude *increase* after FC_AI_ (Figure 3) and latency *decrease (sensitivity increase)* observed in the present study seem to be incompatible with existing data on MOC system activation and suggest that the enhancement of cochlear processing was mediated *via* the lateral olivocochlear system originating in and near the lateral superior olive (LSO). The LSO receives tonotopically arranged descending projections from the ipsi- and contralateral AI (Feliciano et al., 1995; Coomes and Schofield, 2004) and from the ipsi- and contralateral IC (van Noort, 1969; Thompson and Thompson, 1993; Brown et al., 2013), and provides mainly ipsilateral tonotopic projections to auditory nerve-fiber synapses below small sets of cochlear inner hair cells (Warr et al., 1997; Brown, 2011). Local activation of the ipsi- and contralateral IC (ventro-lateral locations) or the ipsilateral LSO could produce slowly starting but long-lasting (more than 20 min) CAP amplitude increases (Groff and Liberman, 2003). LSO lesions either had no effect on CAP thresholds and amplitudes when the stimulating tones had rather low SPLs as in the present study (Darrow et al., 2007) or caused long-lasting decreases of CAP amplitudes, and 0–10 dB increases of CAP thresholds (Le Prell et al., 2003). These evidences suggest that the present FC_AI_ could have increased the sensitivity of the cochlea contralateral to the conditioned AI by about 4 dB and the PI amplitude by 35% through direct or indirect (*via* the IC) activation of the LSO contralateral to the AI. The PI amplitude increase as result of FC_AI_ can be interpreted as a corresponding increase of the excitatory postsynaptic potential amplitudes (summed up as PI amplitude) at the affected inner hair cell–cochlear nerve fiber synapses.

This interpretation of our present data (ABR PI amplitude increase and latency decrease after FC_AI_) assumes a direct LSO augmentation effect on those synapses between inner hair cells and cochlear nerve fibers which processed, according to the cochlear tonotopy, that frequency which was conditioned *via* the FC_AI_. It should be emphasized here that we analyzed rather long-term effects on ABR peaks occurring 1–3 h after the end of auditory cortical conditioning which may basically be different from the immediate and fast effects (time scales of milliseconds, seconds or minutes) on cochlear responses during and after AC, IC or SOC stimulation in other studies (Desmedt and Monaco, 1961; Gifford and Guinan, 1987; Mulders and Robertson, 2000, 2005; Popelar et al., 2002; Xiao and Suga, 2002; Groff and Liberman, 2003; Guinan, 2005; Perrot et al., 2006; Elgueda et al., 2011; Dragicevic et al., 2015; Aedo et al., 2016). Thus, our experimental paradigm allows for plastic changes at the synapses of inner hair cells and cochlear nerve fibers leading to facilitation of these synapses in response to sounds of known importance to the AC. This hypothesis of conditioned frequency-specific facilitation of cochlear responses *via* LSO activation may now be tested in further experiments involving manipulation of cochlear neurotransmission and LSO activity after auditory learning.

The influences of FC_AI_ on CN responses have been shown to be frequency-specific, i.e., CN neurons with BFs very similar to those of the stimulated AI locus increased response rates and decreased response latencies after FC_AI_ (Luo et al., 2008; Liu et al., 2010, 2019; Kong et al., 2014). These enhancements were absent in neurons with unmatched BFs. Our present ABR data on PII, showing significant latency decrease (Table 1) and amplitude increase (Figure 3), are in line with these observations. Interestingly, the latency of CN onset responses decreased only slightly (average 0.17 ms; in Liu et al., 2019) after FC_AI_, corresponding with the small but significant 0.05 ms latency decrease of PII in the present study (Table 1). With the same reasoning as for PI, the 0.05 ms latency decrease can be expressed as 2.25 dB sensitivity increase. The 40% PII amplitude increase (Figure 3A) would reflect a corresponding increase of the excitatory postsynaptic potentials (summed up as PII amplitude) in CN neurons after FC_AI_. This 40% relative amplitude increase of PII was similar to the 34% amplitude increase of PI suggesting that the amount of the corticofugal effect on the CN is comparable with that on the CO. Because of branching of auditory nerve fibers in the CN contacting many neurons in the three CN partitions (e.g., Romand and Avan, 1997) the absolute amplitude increase of PII must be and actually was much larger than in the cochlea (Figure 3). Whether the positive influence of FC_AI_ on excitatory postsynaptic potentials of CN neurons was mediated by direct bilateral projections of the AC to the CN (Weedman and Ryugo, 1996; Jacomme et al., 2003; Schofield and Coomes, 2005) or through multi-synaptic pathways (Malmierca and Ryugo, 2011; Schofield and Beebe, 2018) remains to be clarified. At least, electrical stimulation of the AC could lead to slow excitatory postsynaptic potentials in principle cells of the dorsal CN (Jacomme et al., 2003).

Local electrical stimulation of the mouse AC significantly reduced PIII and PV amplitudes (Aedo et al., 2016) when the ABR was taken immediately after the AC stimulation. The stimulus currents used in that study were 1–4 μA and delivered at a rate of 32 Hz for 5 min. This current density was much higher than that used in the present study (500 nA at 4 Hz for 7 min). ABR PIII amplitude reductions by AC stimulation could be expected *via* activation of the MOC system so that the data reported by Aedo et al. (2016) were interpreted in this way. This means, as explained above in view of our present data, that AC activation may have two different effects on cochlear processing, namely, after strong stimulation immediate and short-term reduction of cochlear and further ascending sensitivity *via* the MOC system and, after conditioning to a given tone, buildup and long-lasting enhancement of cochlear and further ascending processing of that tone *via* the lateral olivocochlear system. As in the CO and CN, the PIII latency decrease by 0.10 ms can be expressed as 4.5 dB sensitivity increase due to FC_AI_, and the increase of the absolute PIII amplitude (Figure 3) as an effect of FC_AI_ on an even larger number of neurons than in the CN. The 50% relative amplitude increase (Figure 3A) was comparable to the relative amplitude increases in the CO and CN suggesting similar amounts of corticofugal effects at the three most peripheral levels of the ascending auditory pathway.

Physiological studies about corticofugal effects on sound processing in the LL seem not to be available. In the study by Aedo et al. (2016), the possible effects on the PIV were not analyzed. The presently observed latency decrease of 0.08 ms (Table 1) suggested a sensitivity increase of 3.6 dB at the level of the LL due to FC_AI_. This sensitivity increase was in the same range as found in the CO, CN and SOC. The relative increase of the PIV amplitude by 78% was, however, much higher (average factor of 1.8) compared to the relative amplitude increases at the levels of the CO, CN, SOC, and IC (Figure 3A). This suggests that the amount of the corticofugal influence on sound processing in the LL was larger compared to CO, CN, SOC, and IC. A simple and direct explanation for this observation is based on the comparison of tone response thresholds of auditory nerve fibers, neurons in the CN and IC of the mouse, and neuronal thresholds in the LL (ventral nucleus) of the rat (mouse data do not exist) with the applied tone level of 45 dB SPL. The neurons stimulated in the AI in the present study had the BFs in the range of 19–32 kHz. Most auditory nerve fibers (Ehret and Moffat, 1984; Taberner and Liberman, 2005), neurons in the CN (Ehret and Moffat, 1984) and in the IC (Ehret and Moffat, 1985; Egorova et al., 2001; Hage and Ehret, 2003) of the mouse had excitatory tone response thresholds well below 45 dB in that frequency range. This means that, when the ABR was taken, virtually all neurons with synchronous responses contributed to the ABR amplitude even when the sensitivity was increased by FC_AI_. In the rat LL, excitatory tone response thresholds ranged from about −10 dB to 70 dB in the central frequency range of hearing (Zhang and Kelly, 2006). Considering such a very broad threshold distribution also for the mouse LL, a sensitivity increase by some dB as the most likely basis of the latency decrease due to FC_AI_ would increase the number of neurons contributing to the ABR amplitude because much more neurons were then stimulated above their threshold. With this explanation, not the amount of corticofugal influence on the LL but the number of receptive neurons would have increased leading to a comparably larger PIV amplitude increase as at the other peaks.

The positive effects of FC_AI_ on PV, representing mainly IC activation in the mouse, were predictable in view of the corticofugal effects on frequency-matched neurons in the IC of bats and mice (decrease of thresholds, increase of response strength; Zhang and Suga, 2000; Zhou and Jen, 2000; Yan and Ehret, 2002; Yan et al., 2005). Interestingly, the FC_AI_-induced shortening of the PV latency (0.17 ms, Table 1) was about 2-fold of those at the lower levels (CO, CN, SOC, LL combined: 0.08 ms), which was equivalent to 7.65 dB decrease in threshold according to the calculation discussed above. The 7.65 dB was surprisingly similar to the 6.2 dB decrease in threshold that we actually measured based on Peaks III/V (Figure 2B). This prediction could be more accurate if the averaged latency decrease at PIII and PV (0.10 and 0.17 ms, Table 1) were used. The close agreement between measured and predicted (from latency measurements) threshold changes allows for the prediction of the changes in ABR threshold from the rise time of the tone amplitude at the start of the tone-burst stimuli without sampling large amounts of data at lower sound intensities.

The latency decrease of 0.17 ms with the predicted threshold decrease of 7.65 dB at PV was a relatively large change in response to FC_AI_ compared to the changes at the lower levels. Since the relative increase of the PV amplitude (49%, Figure 3A) was similar to the amount of relative amplitude increases at the lower levels (except the LL, as explained before), the amount of the corticofugal effect with regard to the percentage of affected neurons in the CO, CN, SOC and IC seemed to be similar. In the IC, however, the corticofugal influence differed between types of neurons belonging to different classes of tuning curve shapes (Yan et al., 2005). FC_AI_ decreased the tone response thresholds of class III neurons (rather symmetrical V-shaped excitatory tuning curves, Egorova et al., 2001) by average 5.57 dB when the BFs of the neurons were matched to the BFs at the respective cortical stimulation locus (Yan et al., 2005). Such a decrease was not observed for IC neurons in the other classes (I, II). The average threshold decrease of 5.57 dB in class III neurons was very similar to the latency-predicted threshold decrease of 7.65 dB at the ABR PV. Therefore, our present ABR data seem to reflect differential effects of FC_AI_ on classes of IC neurons with mainly one class (class III) being responsible for the observed shortening of the PV latency.

In conclusion, we observed, as a pervasive net effect of AI conditioning, a general enhancement of processing at all levels represented by the ABR. Therefore, the proposed core neural circuit of sound-specific auditory plasticity (Xiong et al., 2009) may be extended by a branch reaching down to the auditory brainstem and cochlea. These data included new insights in possible corticofugal modulation of inner hair cell synapses and new corticofugal effects on processing in the SOC and the LL. It will be important in further analysis to clarify whether this corticofugal facilitation of processing sound of “known” importance (to the AC) will turn to inhibition or other changes when the frequency match of stimulation at the AC and lower levels is not given.

## Data Availability

All datasets generated for this study are included in the manuscript.

## Ethics Statement

### Animal Subjects

The animal study was reviewed and approved; the animal protocol was in accordance with the European Communities Council Directive (86/609/EEC) and approved by the appropriate authority (Regierungspräsidium Tübingen, Germany).

## Author Contributions

XL: examining original recording, data analysis and instruct the writing of first draft of this article. OZ: write the first draft of the article. AC and KH: data processing, analysis, statistics. GE: study concept, experimental design, supplies for the experiments, writing the final version. JY: experimental design, experimentation, writing the final version.

## Conflict of Interest Statement

The authors declare that the research was conducted in the absence of any commercial or financial relationships that could be construed as a potential conflict of interest.
